# “If it is left, it becomes easy for me to get tested”: Use of oral self‐tests and community health workers to maximize the potential of home‐based HIV testing among adolescents in Lesotho

**DOI:** 10.1002/jia2.25563

**Published:** 2020-08-31

**Authors:** Alain Amstutz, Mathebe Kopo, Thabo I Lejone, Lefu Khesa, Mpho Kao, Josephine Muhairwe, Tracy R Glass, Niklaus D Labhardt

**Affiliations:** ^1^ Swiss Tropical and Public Health Institute Basel Switzerland; ^2^ University of Basel Basel Switzerland; ^3^ Department of Infectious Diseases and Hospital Epidemiology University Hospital Basel Basel Switzerland; ^4^ SolidarMed, Partnerships for Health Maseru Lesotho

**Keywords:** HIV, adolescent, oral self‐test, Lesotho, HIVST, secondary distribution

## Abstract

**Introduction:**

Home‐based HIV testing fails to reach high coverage among adolescents and young adults (AYA), mainly because they are often absent during the day of home‐based testing. ADORE (ADolescent ORal tEsting) is a mixed‐method nested study among AYA in rural Lesotho, measuring the effect of home‐based secondary distribution of oral HIV self‐tests (HIVST) on coverage, as well as exploring how AYA perceive this HIV self‐testing model.

**Methods:**

ADORE study was nested in a cluster‐randomized trial. In intervention village‐clusters, oral HIVST were left for household members who were absent or declined testing during a testing campaign. One present household member was trained on HIVST use. Distributed HIVST were followed up by village health workers (VHW). In control clusters no self‐tests were distributed. The quantitative outcome was testing coverage among AYA (age 12 to 24) within 120 days, defined as a confirmed HIV test result or known status, using adjusted random‐effects logistic regression on the intention‐to‐treat population. Qualitatively, we conducted in‐depth interviews among both AYA who used and did not use the distributed HIVST.

**Results:**

From July 2018 to December 2018, 49 and 57 villages with 1471 and 1620 consenting households and 1236 and 1445 AYA in the control and intervention arm, respectively, were enrolled. On the day of the home‐visit, a testing coverage of 37% (461/1236) and 41% (596/1445) in the control and the intervention arm, respectively, were achieved. During the 120 days follow‐up period, an additional 23 and 490 AYA in control and intervention clusters, respectively, knew their status. This resulted in a testing coverage of 484/1236 (39%) in the control versus 1086/1445 (75%) in the intervention arm (aOR 8.80 [95% CI 5.81 to 13.32]; *p* < 0.001). 21 interviews were performed. Personal assistance after the secondary distribution emerged as a key theme and VHWs were generally seen as a trusted cadre.

**Conclusions:**

Secondary distribution of HIVST for AYA absent or refusing to test during home‐based testing in Lesotho resulted in an absolute 36% increase in coverage. Distribution should, however, go along with clear instructions on the use of the HIVST and a possibility to easily access more personal support.

## 1 INTRODUCTION

In Southern Africa, adolescents and young adults (AYA) experience high HIV transmission rates as access to HIV prevention, testing and care services remains low among AYA [1, 2, 3, 4]. In 2016, UNICEF estimated that only 13% of adolescent girls and 9% of adolescent boys aged 15 to 19 in Southern Africa have been included in in HIV testing services in the previous 12 months [5].

In high HIV incidence settings, community‐based HIV testing tailored to the needs of AYA are seen as one important pillar to reduce HIV incidence, morbidity and mortality in this age group [4]. However, while uptake is usually at 90% during home‐based testing, the testing coverage remains low due to a large number of household members being absent at the time of the campaign, mainly men, adolescents and young adults [6, 7, 8, 9]. In a previous study from Lesotho, more than 40% of young men aged 15 years and older could not be reached through home visits during week nor weekend days [7]. A promising approach to reach higher testing coverage during home‐based HIV testing may be the use of HIV self‐testing (HIVST). Oral‐fluid rapid HIVST has been shown to increase uptake, particularly among young people, in facility‐ and home‐based testing models across the region [10, 11, 12].

One approach to increase testing coverage using oral HIVST may be its secondary distribution for household members not present during a home‐based testing campaign. The HOSENG (HOme‐based SElf‐testiNG) trial assessed the increase in testing coverage through secondary HIVST distribution to household members, all ages, absent or refusing to test during a home‐based testing campaign in rural Lesotho [13]. Overall, 58% of HIVST distributed were used and returned within 120 days, resulting in an overall HIV testing coverage of 81%, more than 20% higher than in the standard of care arm where no HIVST were distributed [14].

The ADORE (ADolescent ORal tEsting) study is a mixed‐method study nested within HOSENG trial, investigating the effectiveness and perception of secondary oral HIVST distribution with a follow‐up by village health workers (VHWs) among AYA 12 to 24 years old.

## 2 METHODS

### 2.1 Study design, participants and recruitment

The ADORE study is a predefined mixed‐method nested study embedded in the HOSENG trial, a cluster‐randomized trial conducted in 106 rural villages in the catchment area of 20 health facilities of two districts in Lesotho. The randomization was stratified by district (Butha‐Buthe vs. Mokhotlong), village size (≥30 vs. <30 households) and access to the nearest health facility (easy vs. hard to reach, defined as needing to cross a mountain or river or >10 km away from a health facility). An independent statistician was responsible for the computer‐generated randomization list. The study protocol of HOSENG including the nested study ADORE has been published [13]. In short, the trial assessed the increase in HIV testing coverage in intervention clusters through secondary HIVST distribution for household members absent or refusing to test during the day of the home‐based testing campaign compared to control clusters, where no HIVST were distributed during the home‐based testing campaign. Before cluster‐randomization and trial start, the study team obtained verbal consent from all involved village chiefs by attending the village chiefs’ councils and presenting the project. On the day of the HIV testing campaign, the study campaign team, consisting of counsellors and a nurse, obtained a written consent from each household head (or representative aged 18 years or older), to collect household data on all absent and present household members and to propose HIV testing. If the household consented to participate, then the study team enumerated all household members, offered blood‐based HIV testing and counselling plus multi‐disease screening (tuberculosis, alcohol) and HIV prevention (voluntary medical male circumcision referral, condom provision). The counsellors obtained from each household member written informed consent for HIV testing, following national testing guidelines [15]. According to national guidelines individuals aged 12 years or older can consent to HIV testing. For HIVST no written consent was obtained as the act of self‐testing itself represents consent.

In control villages, during the home‐based testing campaign household members with unknown HIV status and absent or refusing to test were encouraged to get tested at the nearest facility.

In intervention arm, study teams offered to leave an oral HIVST (OraQuick^®^) to household members with unknown HIV status and absent or refusing to test if they were 12 years or older. One present household member was trained and tested with the HIVST by a personal step‐by‐step explanation along with the written instruction in Sesotho (the local language). Prior to the campaign, the VHW from intervention villages were trained on the use and interpretation of the HIVST. During the campaign, these VHWs received a list of all household members for whom an HIVST was dispensed and were instructed to visit the households two to four weeks after the campaign to collect the oral HIVST if it was not returned to them before. In the case of a reactive HIVST, the VHW coordinated further blood‐based testing to confirm the outcome.

The qualitative part included 12 to 24 years old participants who refused to use the secondary distributed HIVST and those who used the secondary distributed HIVST, stratified by two pre‐defined factors: male versus female; age 12 to 15 versus 16 to 24 years. They were recruited using purposive sampling, following the concept of saturation [16]. Eligible participants provided written informed consent. Illiterate participants provided a thumb print after a literate witness of their choice read, explained and co‐signed the form, and participants below 18 years chose a caregiver to co‐sign.

This study was approved by the National Health Research and Ethics Committee of the Ministry of Health of Lesotho (ID06‐2018) and the Ethics committee in Switzerland (Ethikkomission Nordwest‐ und Zentralschweiz; 2018‐00283).

### 2.2 Data collection and outcomes

Data collection for the quantitative part of the ADORE study was launched with the start of HOSENG trial on 26 July 2018 in both districts simultaneously. Recruitment lasted for five months. Details about data collection are published elsewhere [13]. The quantitative outcome of ADORE study was testing coverage among AYA (age 12 to 24) within 120 days after home‐based testing, defined as the proportion of all individuals aged 12 to 24 years living in a household of the surveyed area with a confirmed HIV test result. A follow‐up period of 120 days allowed sufficient time for absent members to return to their households, conduct the self‐test, and return it to the VHW. The VHWs re‐read the result of the oral HIVST strip and documented the outcome on a study‐specific form. Furthermore, at all health facilities in both study districts, the study team searched through the testing registers to collect testing outcomes for those participants who decided to come to the clinic for testing instead. Twelve years was chosen as lower age‐limit for oral self‐testing because it is the legal age for providing HIV testing consent in Lesotho [17] and by then available evidence supported the use of the oral HIVST for individuals 12 years of age and older [18]. We defined confirmed HIV test results as (i) known HIV‐positive with documentation (in the health booklet or the national testing register), (ii) known HIV‐negative (tested within previous 4 weeks with documentation in the health booklet or the national testing register), or (iii) confirmed HIV test result during the study period according to the national HIV testing guidelines [15]. We classified a reactive oral HIVST as confirmed only if follow‐up blood‐based testing was performed.

After assessment of the quantitative outcome, one‐on‐one in‐depth interviews were conducted from February 02, 2019 until 14 May 2019, by one trained interviewer. The interviews lasted approximately 20 minutes and were conducted in Sesotho in a private space at participants’ home. The interviewer used a semi‐structured interview questionnaire tailored to participants who refused versus who used the HIVST. The questionnaire covered socio‐demographic characteristics, HIV testing preferences, their individual views on the secondary distribution of HIVST, perceptions about the optimal support needed during usage of HIVST and the follow‐up of distributed HIVST. Two qualitative research objectives were defined:

1. *How do AYA (12 to 24 years old) perceive the use of an oral HIVST that was left for them because they were absent during the day of the HIV testing campaign or because they refused blood‐based HIV testing?*
2. *How do AYA (12 to 24 years old) perceive the involvement of the village health worker in the follow‐up of the distributed oral HIVST?*

### 2.3 Data analysis

The quantitative outcome was analysed following an intention‐to‐treat approach with clusters as unit of randomization and individuals as unit of analysis, using multi‐level logistic regression models including village and household as random effects. The model was adjusted for the pre‐specified randomization stratification factors. Results are presented as adjusted odds ratios (aOR) and 95% confidence intervals (CI). As pre‐defined subgroup analyses, the potential effect modification of sex (male, female) was assessed and intervention effects calculated separately in the case of significant effect modification. All analyses were done using Stata (version 15, Stata Corporation, Austin, TX, USA) and all tests used two‐sided *p*‐values with alpha 0.05 level of significance.

Regarding the qualitative data, audio recordings were translated and transcribed into English, and the analysis process determined according to the Framework Method [19]. A codebook was developed by the qualitative study team, deducting themes and codes from the two qualitative research objectives. Three researchers coded the transcripts independently, line by line and regularly met to compare the coding. In parallel to the rollout of the interviews, a working analytical framework was developed, and the codebook was constantly updated, adding more detailed codes inductively. A matrix was developed to systematically compare relevant responses across participants. Similarities and differences in findings by the stratified groups were identified, and illustrative quotes were selected.

## 3 RESULTS

### 3.1 Quantitative results

#### 3.1.1 Participant characteristics

From July 26, 2018, until December 12, 2018, 49 and 57 villages with 1471 and 1620 consenting households and 1236 and 1445 enumerated AYA in the control and intervention arm, respectively, were enrolled. 843 (68%) AYA in the control arm and 911 (63%) AYA in the intervention arm were absent. Table 1 summarizes the demographic information of all enumerated participants.

**Table 1:** Characteristics of study participants by study arm

	Control	Intervention	Total
N	1236 (100)	1445 (100)	2681 (100)
Absent			
Yes	843 (68.2)	911 (63.0)	1754 (65.4)
No	393 (31.8)	534 (37.0)	927 (34.6)
Reason for being absent			
Work	51 (6.0)	63 (6.9)	114 (6.5)
School	503 (59.4)	466 (50.9)	969 (55.0)
Within the village	109 (12.9)	151 (16.5)	260 (14.8)
Outside the village	166 (19.6)	226 (24.7)	392 (22.2)
Unknown	10 (1.2)	8 (0.9)	18 (1.0)
Other reasons	8 (0.9)	1 (0.1)	9 (0.5)
Age	17.0 (14.0 to 21.0)	18.0 (15.0 to 21.0)	18.0 (15.0 to 21.0)
Gender			
Female	652 (52.8)	776 (53.7)	1428 (53.3)
Male	584 (47.2)	668 (46.3)	1252 (46.7)
Pregnant^a^			
Yes	18 (3.3)	35 (5.0)	53 (4.3)
No	522 (96.7)	662 (95.0)	1184 (95.7)
Main caregiver for the child^b^			
Mother	337 (56.8)	368 (56.1)	705 (56.4)
Father	51 (8.6)	74 (11.3)	125 (10.0)
Other family member	204 (34.4)	213 (32.5)	417 (33.4)
Friend	1 (0.2)	0 (0.0)	1 (0.1)
Neighbour	0 (0.0)	1 (0.2)	1 (0.1)
Orphan^b^			
Yes, single orphan	130 (21.9)	126 (19.4)	256 (20.6)
Yes, double orphan	46 (7.7)	42 (6.5)	88 (7.1)
No	418 (70.4)	481 (74.1)	899 (72.3)
Years of schooling	7.0 (5.0 to 8.0)	7.0 (6.0 to 9.0)	7.0 (5.0 to 9.0)
Work			
Employed in Lesotho	55 (4.5)	79 (5.5)	134 (5.0)
Employed in RSA	17 (1.4)	14 (1.0)	31 (1.2)
Self‐employed	24 (2.0)	28 (2.0)	52 (2.0)
Subsistence farming	71 (5.8)	63 (4.4)	134 (5.0)
No regular income/employment	305 (24.8)	365 (25.5)	670 (25.2)
Housewife	81 (6.6)	121 (8.4)	202 (7.6)
Student	665 (54.2)	753 (52.5)	1418 (53.3)
Child	10 (0.8)	10 (0.7)	20 (0.8)

^a^ Only asked among female participants

^b^ Only asked among participants below 16 years of age.

RSA, Republic of South Africa.

#### 3.1.2 Testing coverage among AYA

**Figure 1:**
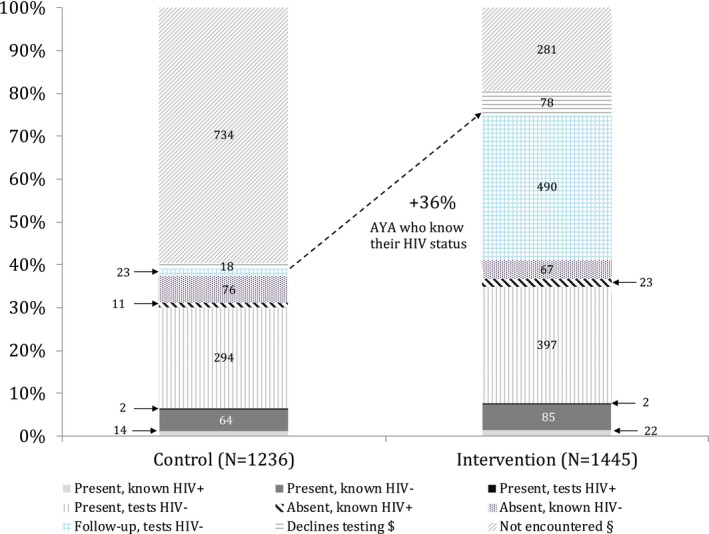
Testing coverage by cluster arm. ^$^They declined testing during home visit and no follow‐up testing outcome available. ^§^They were absent during home visit and no follow‐up testing outcome available. AYA, adolescents and young adults

Figure 1 displays the HIV testing coverage among the study participants in detail. Applying the outcome definitions of a confirmed HIV test results (see above), a testing coverage on the day of the home‐visit of 37% (461/1236) and 41% (596/1445) in the control and the intervention arm, respectively, was achieved. In intervention clusters, overall, 785 oral HIVST were left for AYA who were absent (n = 771) or refused testing (n = 14) during the home‐visit. Uptake of the distributed HIVST was 62% (487/785). At completion of the 120 days follow‐up period after the HIV testing campaign, in the intervention clusters 490 additional AYA – who were initially absent or refused to test during the campaign – knew their HIV status: 99% (487) through usage of the distributed oral HIVST and 1% (3) through testing at a health facility. In the control arm, 23 AYA initially absent or refusing to test attended the health facility for testing within the follow‐up period.

Overall, this resulted in a HIV testing coverage among AYA within 120 days after the home visit of 484/1236 (39%) in the control arm versus 1086/1445 (75%) in the intervention (aOR 8.80 [95% CI 5.81 to 13.32]; *p* < 0.001; Table 2). The intervention effect was greater in male AYA (70% vs. 25%; aOR 16.40 [CI 8.35 to 32.24]) than female AYA (80% vs. 52%; aOR 5.78 [CI 3.55 to 9.41], *p*‐interaction <0.001; Table 2). Linkage to care data will be published separately.

**Table 2:** Quantitative outcome and subgroup analysis

	Control	Intervention	Adjusted odds ratio^a^ (95% CI)	*p*‐value
ADORE quantitative outcome				
HIV testing coverage among AYA^b^	484/1236 (39%)	1086/1445 (75%)	8.80 (5.81 to 13.32)	<0.001
Subgroup analysis on ADORE quantitative outcome				
Gender				
Male	148/584 (25%)	467/668 (70%)	16.40 (8.35 to 32.24)	<0.001
Female	336/652 (52%)	618/776 (80%)	5.78 (3.55 to 9.41)	<0.001

CI, confidence interval; AYA, adolescents and young adults.

^a^ Multi‐level logistic regression models adjusted for clustering (village and household as random effects) and stratification factors (district, village size, and access to health facility as fixed effects)

^b^ Within 120 days of the home visit.

### 3.2 Qualitative results

#### 3.2.1 Interviewee characteristics

Overall 21 participants from intervention village‐clusters were interviewed: 11 who refused to use the HIVST and 10 who did use HIVST. On average, the participants had completed 10 years of schooling, showed moderate HIV/AIDS‐related knowledge and low HIV/AIDS‐related stigma. Over 80% of the participants reported having tested for HIV before (all blood‐based) and 80% in the HIVST user group and 60% in the HIVST non‐user group reported oral‐based testing as preferred test specimen (Table 3).

**Table 3:** Characteristics of interviewees

	HIVST users	HIVST non‐users	Total
N	10	11	21
Age, median (IQR)	15.5 (13 to 17)	15 (14 to 19)	15 (14 to 19)
Female sex, n (%)	5 (50)	7 (64)	12 (57)
Years of schooling, median (IQR)	10 (8 to 12)	9 (8 to 12)	10 (8 to 12)
Single marital status, n (%)	10 (100)	11 (100)	21 (100)
HIV/AIDS‐related knowledge, mean (SD)^a^	5.78 (1.39)^c^	6.09 (1.14)	5.95 (1.23)
HIV/AIDS‐related stigma, mean (SD)^b^	0.56 (0.88)^c^	0.64 (0.81)	0.6 (0.82)
Ever tested for HIV before, n (%)	8 (80)	9 (82)	17 (81)
Prefer blood‐based testing (vs. oral‐based)	2 (20)	4 (40)	6 (29)

^a^ 10 items, 1 point each, the higher the better knowledge, using a validated questionnaire (Bowen et al. BMC Public Health (2016) 16:70)

^b^ 8 items, 1 point each, the higher the more stigma, using a validated questionnaire (Bowen et al. BMC Public Health (2016) 16:70)

^c^ 1 missing data.

#### 3.2.2 Perceptions about secondary distribution of HIVST

Many participants thought secondary distribution was acceptable; “Because if a person wants to test if they were not at home they would miss to test if it was not left” (female, 14 years, HIVST user) Some emphasized that it was a convenient way of getting tested; “I think it's a good thing because I do not go to the doctor, so I do not have many opportunities to get tested so when it is left it becomes easy for me to get tested” (female, 18 years, HIVST user); whereas others pointed out the confidentiality of testing; “I think it's a good thing, because a person is sometimes scared to use while another person is present but if it is left for them that will be easy” (male, 15 years, HIVST user).

A few participants also raised concerns, mainly challenges related to pre‐test counselling. Specifically, some noted that they did not feel ready to test; “I believe I need to think a lot before I can use it, so that I am ready to use the test. No, they did not prepare me in any way” (female, 20 years, HIVST non‐user) or were afraid of the outcome; “I decided not to use it because I am afraid. If I find that I have the infection I would be stressed” (male, 15 years, HIVST non‐user). Others added that no pre‐test counselling happened at all; “I was not able to use it and it was not explained to me how it is used, so I was only told that it is there but I was not explained how it is used when I get home” (female, 15 years, HIVST non‐user).

Both groups were asked about what kind of additional support they would have wished to perform the HIVST that was left for them. A few mentioned better written instructions and adding audio assistance; “That we are left with something to listen to that explains how it is used” (male, 14 years, HIVST user). Someone suggested to hold a public gathering after the distribution; “I recommend that the next time there should be a public gathering, so as to inform people on how to use it, and how it will help them.” (female, 17 years, HIVST user). However, most wished more adequate personal assistance by various personnel; *“*I could have said the village health worker should help me, to help me test for HIV*”* (female, 15 years, HIVST non‐user); “…when it is a nurse who explains to me how this tool is used, and what it is used for” (female, 14 years, HIVST non‐user); “If I had at least got it from the people who have left it or the people from the ministry I believe I could get the right support” (male, 21, HIVST user); “It is that I get assisted by my mother” (female, 16 years, HIVST non‐user).

#### 3.2.3 Views on VHW involvement

Both groups were interviewed about their view to involve the VHW in the follow‐up of secondary distributed HIVST. The participants overwhelmingly expressed positive views. Some participants highlighted that the VHW can offer additional support and counselling; “This is a good idea because the village health worker can also be able to explain to people the use and results to them” (male, 21 years, HIVST user) and clarification; “I think it's good because we're going to make mistakes then she can come back to help and show us we have to do this and that” (female, 18 years, HIVST user). Others emphasized the aspect of confidentiality; “Yes [the VHW] could pick them up, it's best because if it is one of those in our teens or young people they are likely to look at others but if it is an elderly person it is much better” (female, 20 years, HIVST non‐user) and convenience; “Yes, [the VHW can] take them back to the people who distributed them” (male, 13 years, HIVST non‐user).

However, one participant expressed major concerns. She argued that the VHW could disclose the status of the tested person, thus leading to discrimination in the village;

“To give to them? No. because in the village as I previously mentioned when a person has tested themselves and discovers that they are positive, since they will have to take it to the village health worker, they might think that when he meets you on the road here they may humiliate or tell other people, and then you will end up living in fear without self‐esteem.” (female, 20 years, HIVST non‐user)

## 4 DISCUSSION

In sub‐Saharan Africa HIV transmission remains disproportionally high among AYA. [4] Many national programmes for HIV testing appear not to address enough the needs and demands of AYA, resulting in low testing coverage and delayed access to care. [20] Using quantitative and qualitative methods, the ADORE study contributes to the literature on how to better reach AYA during home‐based testing in rural Lesotho: Secondary distribution of oral HIV self‐tests and subsequent follow‐up by a VHW for AYA, who were initially absent or refused to test during a testing campaign, increased testing coverage within a period of four months by 36%. The existence of a long‐standing VHW network in Lesotho – similar to many other sub‐Saharan African countries – makes this model feasible and scalable with little additional costs. Findings from the qualitative research suggest that AYA perceived oral HIVST as a convenient and confidential way for getting tested, and that in general, VHWs appear to be a trusted lay cadre for the follow‐up of HIVST.

As a caveat, even though distribution of oral HIVST increased testing coverage among AYA to 75%, it still fell short of the targeted 90% coverage, and 38% of the AYA who received a HIVST through secondary distribution did not use it. As stated during the in‐depth interviews, a few AYA felt uncomfortable in doing the test alone, others stated, generally not being ready to test as they were afraid of the result. During the 4‐month follow‐up no HIV‐positive test result was recorded. We may have to assume that a number of individuals who had a reactive HIVST result did not bring the test kit back to the VHW or the facility – at least not within the set outcome window. These caveats emphasize the importance of differentiated service deliveries for AYA – in line with current policies from international HIV agencies and the WHO [21, 22].

Direct (i.e. primary) distribution of HIVST in sub‐Saharan Africa has shown to successfully reach high testing rates among AYA across various testing modalities [23, 24, 25, 26]. However, to our knowledge, ADORE is among the first studies exploring secondary distribution of HIVST during home‐based HIV testing in AYA. A nested trial within HPTN 071 (PopART) investigated primary as well as secondary HIVST distribution during a door‐to‐door testing campaign [27]. This led to a moderate increase in coverage rates from 65% to 68% overall, and from 70% to 74% among young adults aged 16 to 29 years old. The authors, however, were not able to assess if the positive effect on coverage was driven by the primary or secondary distribution.

The benefit of secondary oral HIVST distribution in ADORE study was particularly high among male AYA, although overall testing coverage achieved remained below the coverage rate of female AYA. Among male AYA, testing coverage increased from 25% to 70% (Table 2). Given the generally lower access to HIV testing among men, particularly young men, this finding encourages national programmes to include secondary distribution of oral HIVST into any community‐based HIV testing campaign. The PopArt nested study reported similar findings but with different effect sizes: By distributing HIVST in the intervention arm, the coverage significantly increased by 6% among male AYA, but only by 1% among female AYA [27]. The gender difference may be driven by the fact that more male AYA than female AYA are usually absent during home‐based HIV testing [7]. In our study male AYA made up 57% (991/1754) of all absent household members. On the other hand, a recent cross‐sectional study from the Democratic Republic of the Congo assessed preferences of HIV testing among 600 adolescents and concluded that home‐based HIVST was preferred over facility‐based testing, especially among male adolescents [28]. The fact of having more control over the testing process thanks to self‐testing is particularly appealing to young men and may explain high uptake of testing [29].

During the global roll‐out of HIVST concerns have been raised that HIVST may lead to unintended social harm. However, a systematic review [30], as well as a large‐scale assessment within the STAR initiative [31] found little evidence to support this concern. During the follow‐up of HIVST our VHW did not report any serious adverse events related to HIVST, nor did our interviewees.

Rather surprisingly, in our study interviewed AYA were almost unanimously in favour of integrating the VHW in the post‐test process, which may indicate that the engagement of VHW in the HIV/AIDS response is acceptable for AYA in this setting. This is an encouraging finding in light of the UNAIDS 2017 initiative to recruit two million African community health workers ensuring an effective and sustainable response to the HIV/AIDS epidemic in Africa [32]. On the other hand, universal home‐based testing may become less frequent in future as its yield of new HIV diagnoses has become extremely low and donors may reduce funding for such approaches.

Our study has several limitations. First, calculation of HIV testing coverage considered only those who either had a proof of recent testing (within the last four weeks) or tested within the study. Some individuals, particularly among those absent, may have tested for HIV at a different occasion or at facilities outside the study districts. Second, some may have used the test but did not return it to the VHW. Both factors would lead to an underestimation of the actual testing coverage. Third, purposive sampling for the qualitative interviews may have resulted in an interviewee population generally more open to healthcare services than individuals who could not be reached for or refused interviews. On the other hand, half of the interviewed participants did not make use of our intervention and thus may have contributed to a comprehensive picture. Overall, the qualitative data has insufficient depth with only 21 interviews but may still give a hint why the intervention worked. Fourth, a more extensive interview design would have been needed to thoroughly explore social harm of our intervention. Fifth, due to the design of the study we were not able to explore other follow‐up methods after HIVST usage than by the VHW. Future research should investigate the optimal training of the present household member as well as the VHW, include phone numbers of nearby health personnel and VHW that can assist, and explore new technologies, such as audio and video instructions, for conducting the follow‐up after secondary HIVST distribution.

## 5 CONCLUSIONS

The ADORE study, conducted in Lesotho, Southern Africa, shows that secondary distribution of oral HIVST for AYA absent or refusing to test during home‐based HIV testing results in an absolute 36% increase in testing coverage. Based on these findings we encourage secondary HIVST distribution for AYA who cannot be reached during testing campaigns. Secondary distribution should, however, go along with clear instructions on the use of oral HIVST and a possibility for AYA to easily access support if they wish so. In our study, village health workers who are mainly older female members of the community appear to be a trusted cadre for the follow‐up of distributed HIVST among AYA, as long as confidentiality is ensured.

## COMPETING INTERESTS

The authors declare that they have no competing interests.

## AUTHOR’S CONTRIBUTIONS

AA, JM, MK, NDL, TIL and TRG conceptualized and designed the study. AA and NDL drafted the first version of the manuscript. MK is the study coordinator and conducted the interviews. LK and MKa coordinated quantitative data collection. AA, MK and TIL analysed qualitative data. TRG analysed quantitative data. JM provided technical support. All authors read, critically revised and approved the final manuscript.

## ABBREVIATIONS

AYA, Adolescents and Young Adults; ADORE, ADolescent ORal sElf‐testing; CI, Confidence Interval; HIV, Human Immunodeficiency Virus; HIVST, HIV Self‐Test(ing); HOSENG, HOme‐based SElf‐testiNG; IQR, Interquartile Range; OR, Odds Ratio; VHW, Village Health Worker; WHO, World Health Organization.
